# Design and Characterization of a Novel ZnO–Ag/Polypyrrole Core–Shell Nanocomposite for Water Bioremediation

**DOI:** 10.3390/nano11071688

**Published:** 2021-06-28

**Authors:** Fatma Mohamed, Abeer Enaiet Allah, Khulood A. Abu Al-Ola, Mohamed Shaban

**Affiliations:** 1 Nanophotonics and Applications (NPA) Lab, Physics Department, Faculty of Science, Beni-Suef University, Beni-Suef 62514, Egypt; fatma.mohamed@science.bsu.edu.eg; 2 Chemistry Department, Faculty of Science, Beni-Suef University, Beni-Suef 62511, Egypt; abeer.abdelaal@science.bsu.edu.eg; 3 Department of Chemistry, College of Science, Taibah University, Al-Madinah Al-Munawarah 30002, Saudi Arabia; kabualola@taibahu.edu.sa; 4 Department of Physics, Faculty of Science, Islamic University in Madinah, Al-Madinah Al-Munawarah 42351, Saudi Arabia

**Keywords:** ZnO–Ag/polypyrrole nanocomposite, core–shell, electrical properties, antimicrobial activity, adsorption, bioremediation of water

## Abstract

Incorporating nanostructured metal and metal oxide in a polymer matrix is a strategic way to develop a novel candidate for water bioremediation. In this study, under microwave irradiation, a ZnO–Ag/polypyrrole (PPy) nanocomposite with a core/shell structure was prepared by interfacial polymerization of pyrrole in the presence of ZnO nanoparticles and AgNO_3_ as an oxidant. The antimicrobial behavior of the ZnO–Ag core combined with the electrical properties of the conducting PPy shell created a special ZnO–Ag/PPy nanocomposite with inherent adsorption behavior and antimicrobial properties. More impressively, the as-prepared ZnO–Ag/PPy displayed enhanced adsorption of Cd^2+^ and PO_4_^3−^ ions in the mixed solution. At pH 8, it had overall removal efficiencies of 95% and 75% for Cd^2+^and PO_4_^3−^ ions, respectively. The Freundlich adsorption model, rather than the Langmuir adsorption model, better fits the adsorption isotherm results. The adsorption kinetics also followed the pseudo-second-order kinetic model. Additionally, the engineered nanocomposite demonstrated antifungal activity against different fungi, as well as remarkable antibacterial activity against Gram-negative and Gram-positive bacteria. The synergistic combination of crystallinity, coherence of the ZnO–Ag core in the PPy matrix, and the negative zeta potential all contribute to this nanocomposite’s high efficiency. Our results have significant consequences in the wastewater bioremediation field using a simple operation process.

## 1. Introduction

The spread of endemic diseases across the world is becoming a major issue. To date, wastewater produced by industrial development is the primary source of these diseases. Wastewater is any water that has had its original content degraded by adding heavy metals, bacteria and microorganisms, organic and inorganic chemicals, organic contaminants, oils, and other compounds that alter its true properties. Heavy metals and oxyanions are the most common pollutants globally, originating from both industrial and anthropogenic activities [1,2,3,4,5].

Phosphate is an important resource for many species since it is one of the most important contributors to the eutrophication process in water. As a result, the marine ecosystem suffers from severe water quality degradation due to dissolved oxygen depletion, resulting in environmental issues [6,7]. In diffusing into the groundwater and the soil, hazardous heavy metal contamination in wastewater poses a major risk to humans [2]. Owing to its nonbiodegradability, toxicity, and dangerous nature, cadmium is one of the most harmful heavy metals to human health. It can be found in natural, commercial, and agricultural settings [8,9]. Microbial pollution in water is often hazardous to one’s health and can lead to spreading infectious diseases. A bacterium known as methicillin-resistant *Staphylococcus aureus*, abbreviated as S. *aureus*, is one of the most violent but natural human pathogenic agents. It is one of the most common nosocomial pathogens that resist different forms of antibiotics [10,11]. Several methods were used to remove these toxins from wastewater, including physical and material precipitation, particle exchange, reverse osmosis, layer filtration, electrochemical treatment, and dissolvable extraction [4,5]. Any of these methods have the disadvantage of being expensive and requiring vast quantities of chemicals. Others have poor selectivity when competing anions are present. Due to its simplicity and ease of operation, adsorption has recently become a common and widely used process for water remediation. The effectiveness of that technique is determined by selecting an appropriate sorbent in terms of quality and cost [5,12,13].

Recently, conducting polymers have attracted considerable scientific interest in various field applications, such as microelectronics, optoelectronics, biosensors, antimicrobial agent, and adsorbents [14]. Polypyrrole (PPy) conducting polymeric-based adsorbent is the most extensive applied polymer among the various conducting polymers. It exhibits a major role during the adsorption process of different heavy metal ions due to their easy synthesis, high chemical stability, inexpensiveness, and nontoxicity [15,16,17]. The excellent adsorption prospect of PPy is attributed to the presence of positively charged nitrogen atoms of the amine group in the polymeric backbone, which can bind transition metal ions because of its strong affinity towards the divalent metal ions [18]. However, pure PPy tends to aggregate during synthesis into irregular agglomerate morphology by the strong π-π* interactions between the main pyrrole chain, resulting in marginal physical properties, insolubility, and low adsorption capacity, which limits its use [19]. To overcome these hurdles, the fabrication of synthetic PPy nanocomposites is expected to be the most effective modification method for PPy. Currently, reinforcement the polymeric matrix by incorporating fillers, such as carbon materials, natural clays, layered silicate, metal, and metal oxide nanoparticles [20,21,22,23]. Nowadays, polymer-inorganic hybrid nanocomposites have attracted substantial attention because of the possibility of combining the properties of organic and inorganic components for innumerable applications in various fields. Conducting polymer PPy with metal M (PPy/M) or metal oxide MO (PPy/MO) composites have emerged as an attractive material for wastewater treatment due to the interesting synergistic effect between polymer and metal [21,24]. PPy/silver composites were prepared by a single-step method via oxidative chemical polymerization of pyrrole using silver nitrate as an oxidant in an aqueous medium at room temperature with enhanced antimicrobial activity [20,25,26]. Different shapes of PPy/Ag nanoparticles NPs have been fabricated as core/shell structure, nanorod, and nanofibers in previous papers [27,28]. Ag NPs have attracted great interest in the literature due to their nontoxicity, excellent biocompatibility, high adsorption capacity due to large surface area to volume ratio, high antimicrobial properties, and reusability [19,29]. On the other hand, PPy as a conductive polymer with metal oxide nanocomposites has brought out more fields of water treatment application. Zinc oxide (ZnO) nanoparticles (NPs) have been investigated as an efficient adsorbent nanostructure due to their environmentally friendly, high surface area, high catalytic efficiency, nontoxicity, biocompatibility, and chemical stability [30]. Hence, many studies reported the bioremediation effect of ZnO and Ag NPs for wastewater treatment [31,32]. Ahmed et al. reported synthesizing polyaniline zinc oxide nanocomposite by oxidative free-radical polymerization in a multistep reaction. The prepared composite was applied to removing Cr(VI) from aqueous solutions with high adsorption capacity [23]. Moreover, ZnO nanoparticles (NPs) showed good antimicrobial activity against various kinds of bacteria, including both Gram-positive and Gram-negative bacteria [19,30,33,34]. Consequently, it is significant to address the design of the ZnO–Ag/PPy nanocomposite and study its response to adsorbing inorganic pollutants (in particular, Cd^2+^ and PO_4_^3−^ ions) from wastewater and its antimicrobial properties. Compared with conventional preparation methods, microwave synthesis, as a novel heating model, has generated wide interest in nanocomposite fabrication due to some advantages, such as rapid heating, homogeneous thermal transmission, and shorter reaction time [15,35]. In addition, this technique is distinguished by its ability to regulate properties, repeatability, reproducibility, short synthesis time, low cost, purity, and environmental friendliness [36].

This work aims to gain insight into the synergistic interaction between the polymer and metal oxide for water bioremediation. As proof of concept, the preparation of a low-cost ZnO–Ag/PPy nanocomposite with core/shell structure by microwave irradiation technique is achieved. The concept is based on forming the PPy shell on the core structure of Ag and ZnO. The prepared ZnO–Ag/PPy demonstrated efficient removal of the inorganic pollutants (Cd^2+^ and PO_4_^3−^ ions) from wastewater in single and binary contaminants aqueous systems. Moreover, it showed antimicrobial activities against fungi, Gram-positive and Gram-negative bacteria.

## 2. Materials and Methods

### 2.1. Materials

Pyrrole (C_4_H_4_NH, ≥98%, Sigma Aldrich, Munich, Germany), Zinc sulfate (ZnSO_4_, ≥98%, Sigma-Aldrich, Munich, Germany), hydrochloric acid (HCl, ≥37%, Sigma-Aldrich, Munich, Germany), cadmium nitrate (Cd(NO_3_)_2_, ≥98%, Sigma-Aldrich, Munich, Germany) and sodium hydroxide pellets (NaOH, N98%, Sigma-Aldrich, Munich, Germany), sodium dihydrogen phosphate (NaH_2_PO_4_.2H_2_O, 99.95%, Sigma-Aldrich, Munich, Germany), potassium persulfate (K_2_S_2_O_8_, 99.99%, Sigma-Aldrich, Munich, Germany), silver nitrate (AgNO_3_, ≥99%, Sigma-Aldrich, Munich, Germany) and chloroform (CHCl_3_, 99%, Sigma-Aldrich, Munich, Germany) were purchased and used. All the reagents used are of AR grade and used as received.

### 2.2. Synthesis of ZnO NPs

Briefly, a 0.2 M aqueous solution of ZnSO_4_ was prepared in a beaker with 50 mL of deionized water (DW) under continuous stirring at 500 rpm for 20 min at room temperature. In another beaker, a 0.4 M aqueous solution of NaOH was mixed with 50 mL of DW. Then, the NaOH solution was added drop wisely to the ZnSO_4_ solution under continuous stirring for 18 h. A white precipitate can be seen and separated by filtration and washing with distilled water before drying at 60 °C for 12 h. The formed ZnO NPs were calcined at 300 °C using a muffle furnace for 6 h.

### 2.3. Synthesis of the ZnO–Ag/PPy Composite

First, 0.1 M pyrrole was dissolved in 20 mL of CHCl_3,_ and 0.2 g ZnO NPs were added to the solution. Then, 1.06 g of AgNO_3_ was dissolved in 20 mL of distilled water. Subsequently, pyrrole and AgNO_3_ solutions were mixed, and this reaction mixture has proceeded at the H_2_O/CHCl_3_ interface under microwave radiation of 900 Watt for 3 min. A general domestic microwave of Black and Decker 30 L microwave oven MZ3000PG (B. TECH, Cairo, Egypt) with 800 watts was used to carry out the reaction. After this, the complete mixture was filtered. The filtrate was separated using a separating funnel. The filtered product was again washed with distilled water and methanol to remove unreacted Ag^+^ ions. Finally, the product was dried at 60 °C for 12 h.

### 2.4. Adsorption of Cd^2+^ and PO_4_^3−^ Pollutant Ions in Single and Binary Contaminates System Solutions

All batch experiments of equilibrium adsorption in single and binary contaminants system solutions of Cd^2+^and PO_4_^3−^ on nanocomposite adsorbent were conducted at room temperature (25 °C). The specified amount of prepared adsorbent ZnO–Ag/PPy composite was placed into series of Erlenmeyer flasks contain Cd^2+^and PO_4_^3−^ single solutions. Then, to obtain a binary contaminant system, Cd^2+^ and PO_4_^3−^ ions solutions with concentrations 12 and 20 mg/L, respectively, were mixed. The flasks were placed on an orbital shaker and shaken at 200 rpm for 24 h. Subsequently, after filtration through 0.22 μm filter paper, the precipitates were separated from metal ion solution. The concentrations of the metal ions solution before and after adsorption were measured by atomic absorption spectrometry AAS (Agilent Technologies 200 Series AA, Santa Clara, CA, USA) and UV/Vis spectrophotometer (UV-2600i, Shimadzu, Columbia, MD, USA). Herein, the influences of different factors, such as pH (2−10), nanocomposite dose in the range 0.025–0.1 g/L, and reaction time from 1 h to 24 h, were optimized to achieve the maximum removal ability of the nanocomposite. All the experiments were carried out in triplicate to avoid any discrepancy in experimental and to ascertain their reproducibility. The average concentration was determined using SPSS version 16. Results were confirmed by using mean and standard deviation (±SD) values. The results were considered statistically significant at *p* values less than 0.05.

Based on the following equations, the adsorption efficiency of metal ions onto the nanocomposite, Q, equilibrium adsorption capacity, q_e_ (mg/g), were estimated by Equations (1) and (2), respectively:

(1) $$Q=\frac{\left(C_{o}-C_{t}\right)}{C_{o}}\times 100$$

(2) $$q_{e}=\frac{V}{W}\left(C_{o}-C_{e}\right)$$

where C_o_ represents the initial metal ion concentration, C_t_ is the concentration of ions (mg/L) after adsorption time t (min), C_o_ refers to the initial concentration of pollutants ions before adsorption in mg/L, C_e_ is the equilibrium concentration of pollutants in mg/L fixed at 24 h to achieve equilibrium, V is the volume of metal ions solution in a liter, and W refers to the adsorbent weight in gram.

### 2.5. Chemical Composition, Morphological, Structural, and Electrical Properties

The structural properties were investigated using a PANalytical (Empyrean, Malvern Panalytical, Malvern, UK) X-ray diffractometer (Cu Kα, 0.154 nm^−1^) at 40 kV, 35 mA, and scan step 0.02° in the range from 20 to 70°. The average crystallite size (D) of prepared materials was calculated according to the Debye–Scherer relation (D = 0.9λ/Wcosθ), where W is the full width at half maximum in radians, θ is the Bragg’s angle, and λ is the X-ray wavelength (CuKα = 0.15405 nm) [37]. The FT-IR spectra of the ZnO NPs and the prepared nanocomposite were recorded with a Bruker Vertex 70 FTIR-FT Raman (Bruker, Ettlingen, Germany) spectrometer. The nanomorphologies and chemical compositions of the nanocomposite were characterized using a transmission electron microscope (TEM, JEOL-JEM 2100, JEOL, Tokyo, Japan) and field-emission scanning electron microscope (FE-SEM, Gemini, Zeiss-Ultra 55, Carl Zeiss SMT Ltd., Cambridge, UK). Particle size and zeta potential analyzer are used to determine particle size distribution, particle zeta potential (related to the magnitude of the electrical charge at the particle surface), and molecular weight of large polymeric substances dispersed in water. The hydrodynamic diameter and zeta potential values of the nanocomposite produced were evaluated using a Malvern zeta sizer nanosystem (Worcestershire, UK) at room temperature (25 °C). Therefore, an aqueous suspension of the ZnO–Ag/PPy composite was made by dispersing 0.01 g/L of composite in a 10 mM NaCl aqueous solution and sonicating it for 30 min before filtering it using vacuum filtration. The size of the distributed nanocomposite was measured using the principle of dynamic light scattering (DLS) technique (Malvern Instruments Ltd., Malvern, UK). For stability determination, solutions were diluted at least 5 times with ultrapure water to obtain reliable particle size measurements. The electrical properties and dielectric constant measurements were addressed over a frequency range up to 5 MHz at different temperatures ranging from 283–298 K using the LCR bridge (EUCOL U2826, Changzhou, China).

### 2.6. Antimicrobial Measurements

Whatman filter paper disks were made using standard size (50 mm width) and held in 10 screws topped wide-mouthed containers to ensure disinfection. The jugs were then placed in an oven set to 150 °C. Following this, the standard plates of the cleaned filter paper were provided with the nanocomposite in DMSO (1 mg/mL). Then, they’ve now seeded a supplement agar plate with the requisite test species in triplicate. The standard conditions of 10^6^ colony-forming units/mL (CFU/mL), then 10^4^ CFU/mL were utilized for antibacterial evaluation. Every plate was inoculated with two filter paper disks. The test organisms were *Pseudomonas aeruginosa* (RCMB 010,043) and *Klebsiella pneumonia* (RCMB 01002 23-5) as examples of Gram-negative bacteria; *Bacillus subtilis* (RCMB 010,067) and *methicillin-resistant Staphylococcus aureus* (MRS A, RCMB 01001 94–5) as examples of Gram-positive bacteria; *Geotrichum candidum* (RCMB 05096) and *Candida albicans* (RCMB 0925) as examples of fungi. Gentamycin, ampicillin, vancomycin, and amphotericin B were utilized as antibiotic references against Gram-negative bacteria, Gram-positive bacteria, MRSA, and fungi. DMSO was tested for antimicrobial activity against the provided microorganisms and showed no antimicrobial activity. The plates were incubated at 37 °C for 24 h and 28 °C for 48 h for bacteria and fungi, respectively. The derivative showed a substantial growth inhibition zone via the twofold serial dilution technique reference.

### 2.7. Minimal Inhibitory Concentration (MIC) Measurement

The antimicrobial activity was assessed using a microdilution powerlessness trial in Muller–Hinton broth (Oxoid) and Sabouraud liquid medium (Oxoid). In DMSO, the stock-tested compound solutions were produced. The standard method broth (Difco) was then used to dilute the stock solution. This led to the preparation of serial twofold dilutions of the broth containing around 10^6^ CFU/mL of test bacteria. Each dilution was then added to a well of a 96-well microtiter plate. The subsequently sealed microplates were incubated at 37 °C for 24 h in a humid chamber to observe the antibacterial activity. Before the incubation period was completed, values of MICs were recognized as the lowest concentrations of the substance that had no visible turbidity. The experiment’s blank was DMSO, and uninoculated media were run parallel with the test compounds under the same conditions. To ensure that the tests were repeatable, they were carried out in triplicate. Using SPSS version21, mean and standard deviation (SD) values were calculated, and *p* values less than 0.05 were considered statistically significant.

## 3. Results and Discussion

### 3.1. Characterization of the Fabricated Nanocomposite

#### 3.1.1. Structural Properties and Chemical Composition

**Figure 1:**
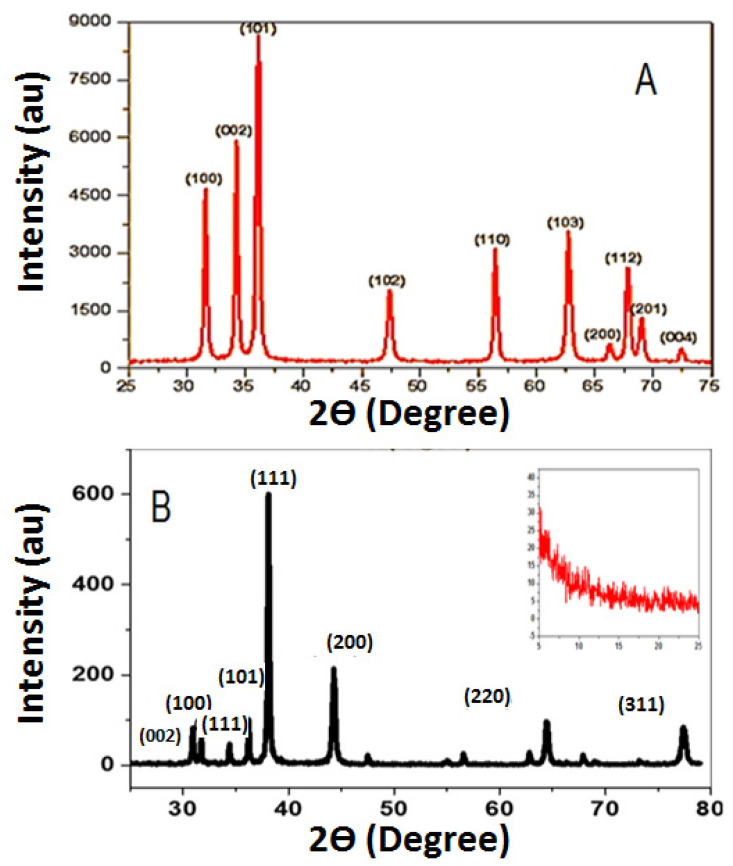
XRD patterns of ZnO NPs (**A**) and ZnO–Ag/PPy nanocomposite (**B**). Inset shows a broad peak of PPy.

XRD patterns of ZnO NPs and nanocomposite were investigated and shown in Figure 1. The characteristic diffraction peaks of pure ZnO NPs in Figure 1A were detected at 2θ of 31.67°, 34.31°, 36.14°, 47.40°, 56.52°, 62.73°, 66.28°, 67.91°, 69.03°, and 72.48°. These peaks are corresponding to (100), (002), (101), (102), (110), (103), (200), (112), (201), and (004) planes of ZnO NPs. These peaks are in good agreement with the characteristic peaks of the polycrystalline wurtzite ZnO structure (Zincite, JCPDS 5-0664). [37]. The obtained value of the average crystallite size of ZnO NPs was ~22 nm. In Figure 1B, the characteristic diffraction peaks of the ZnO–Ag/PPy nanocomposite displayed a broad peak about 2θ = 10°, indicating that the PPy inside the nanocomposite matrix is highly amorphous. Furthermore, it displayed intense diffraction peaks at 2θ = 31.7°, 34.3°, 36.2°, 47.5°, and 56°. i.e., the hexagonal crystalline ZnO NPs embedded in the composite have peaks corresponding to (100), (002), (101), (102) and (110) lattice planes, with the preferred peak at 2θ = 36.2°, and a d-spacing of 2.48 Å [37]. For the face-centered cubic (fcc) structure of Ag nanoparticles, narrow and extreme diffraction peaks were observed at 38.2°, 44.4°, 64.4°, and 77.3° due to Bragg’s reflections from (111), (200), (220), and (311) lattice planes, respectively [38]. The average crystallite size of Ag is equal to ~40 nm [37].

The elemental composition of the composite was examined by the energy-dispersive X-ray (EDX) spectroscopy, Figure S1 (Supplementary Data). It revealed the presence of C (63.8%), N (8.87%), Ag (8.02%), Zn (2.2%) and O (17.1%) signals in the composite. This confirms the high purity of the prepared composite because no impurity signals are detected, which matches the XRD results.

#### 3.1.2. Morphological Properties

**Figure 2:**
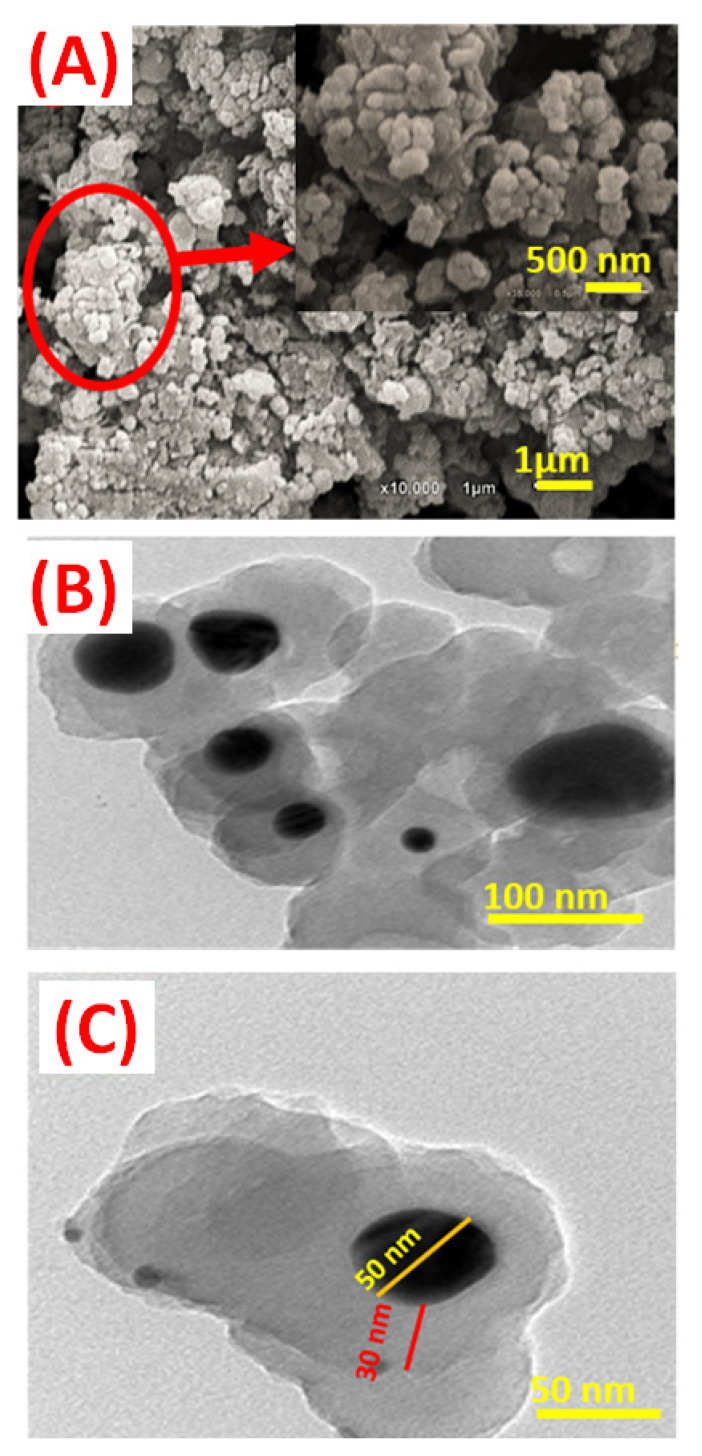
(**A**) SEM, (**B**,**C**) TEM images of the ZnO–Ag/PPy nanocomposite.

The morphology of the ZnO–Ag/PPy nanocomposite was observed by FE-SEM and HRTEM, as shown in Figure 2. The nanoparticles formed as aggregates of near spheres with no crystalline habit that self-assembled to cover the surface. As shown in the FE-SEM image in Figure 2A, the particle size distribution was obtained and is presented in the histogram of Figure S2 (Supplementary Data). The particle size distribution ranged from 52 to 78 nm. The average value was ~75 nm according to the Gaussian fitting. This particle size was greater than the crystallite size obtained from XRD. This suggests that the apparent SEM particles may have consisted of more than one XRD crystallite. HRTEM micrographs of the ZnO–Ag/PPy, Figure 2B,C, showed a high degree of coherence of the ZnO–Ag nanoparticle core in the PPy matrix as a shell, forming the core/shell structure of the ZnO–Ag/PPy nanocomposite. The nanocomposite contained a sheltered black core of the ZnO–Ag nanoparticles with a diameter of about 50 nm and a light-colored cloudy shell of PPy matrix with a thickness of about 30 nm. The HRTEM results also suggest that nanocomposite developed via the interfacial polymerization of pyrrole in a water/chloroform interface in the presence of ZnO nanoparticles and AgNO_3_ as an oxidant using the microwave irradiation technique. The reaction of pyrrole with silver nitrate is considered a redox reaction. The pyrrole monomer acts as a reducing agent due to the presence of the basic –NH group, while the initial silver nitrate acts as an oxidizing agent. In addition, the standard reduction potential of silver and the standard oxidation potential of pyrrole (0.8 V vs. NHE) are ordered in the same region. The formation of silver metal could be explained through the promotion of the silver ions during the polymerization of the pyrrole, generating the polypyrrole (PPy). Silver ions limited the quantity of produced polypyrrole to equal to or less than the number of silver ions available for polymerization. Finally, the excess of pyrrole completely reduces the silver ions to zero-valent silver, forming silver nanoparticles. Moreover, their nucleation and growth in the core ZnO are protected by the formed PPy shell by interfacial polymerization of pyrrole via H_2_O/CHCl_3_ interface, which eventually results in forming the ZnO–Ag/PPy nanocomposite [17,29]. In addition, microwave radiation initiated the oxidation for forming PPy. A longer reaction time was required to break AgNO_3_ for forming Ag nanoparticles, resulting in forming the ZnO–Ag/PPy composite. A schematic representation for forming Ag nanoparticles is shown in Scheme S1 (Supplementary Data). Furthermore, in the PPy matrix, NPs were evenly distributed. Little or no- aggregation of the Ag nanoparticles in the PPy, indicating that the current approach was advantageous over other reported methods.

**Figure 3:**
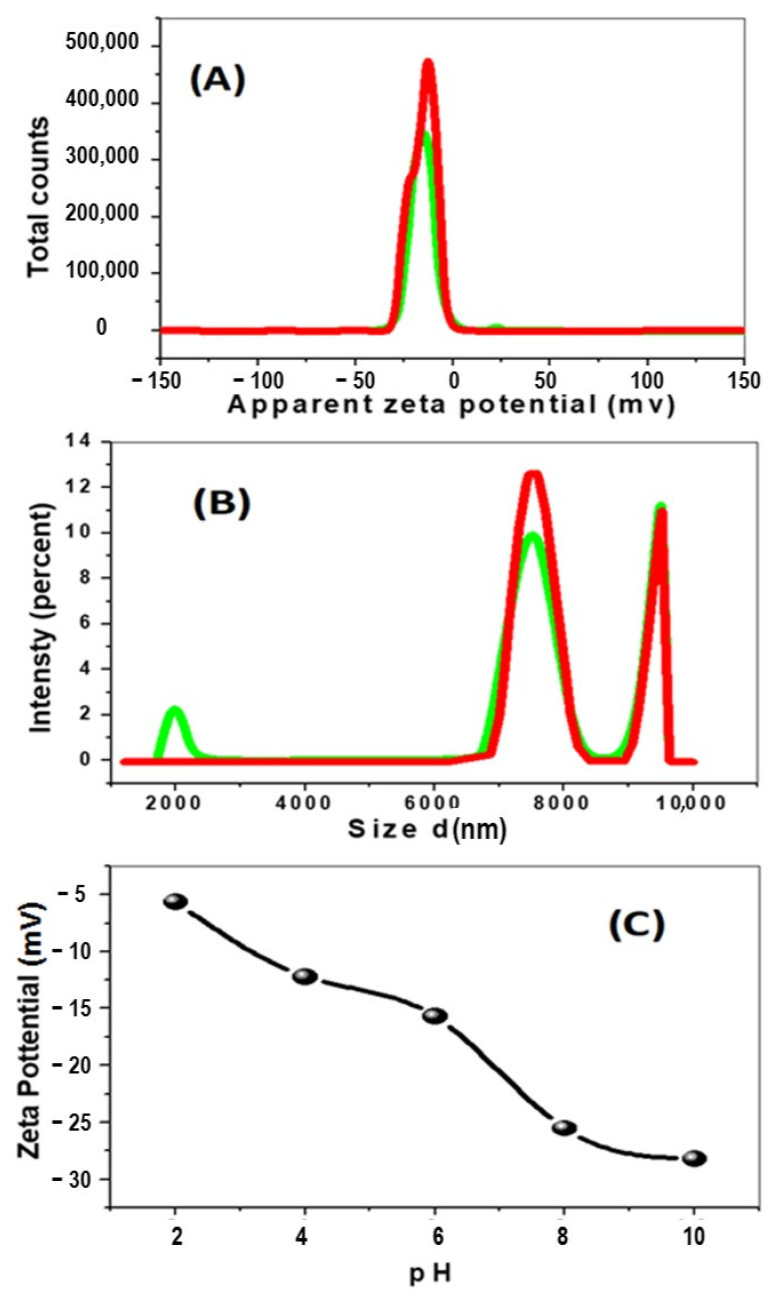
Zeta potential (**A**), particle size (**B**) of the ZnO–Ag/PPy nanocomposite, and (**C**) the effect of pH value on zeta potential.

The zeta potential is a key character for many applications, including the characterization of polymers. The isoelectric point (IEP) is the pH of a compound solution at which the net charge or its zeta potential is zero. The used experimental method can change the value of the isoelectric point due to the measurement techniques. Therefore, there are several different values of IEP for the same ZnO in the literature. For example, IEP values of ZnO were found at pH 8.0, 9.7, 9.8, and 10.3 [39]. The size comparison showed that the solutions with pH less than 3 and greater than 7 have smaller nanoparticles, whereas, in the pH range 3–7, the Ag nanoparticles have a larger size, which is consistent with a possible aggregation of the nanoparticles. The pH is directly related to the stability of the nanoparticles. The change in pH can alter the double-layer properties that can directly influence the zeta potential of the system, making the chances of flocculation or coagulation because each type of nanoparticles is stable near the isoelectric point. AgNPs exhibited an effective charge change in aqueous conditions, from positive (in low pH) to negative (at high pH), with an isoelectric point between them, in which it presented a smaller size value [39]. After modifying another compound as polypyrrole, the composite becomes negatively charged over the whole examined pH range. It was obvious that the pH dependence of zeta potentials for ZnO nanoparticles shifted completely after forming the core–shell structure [40]. The nanocomposite’s zeta-potential as a function of pH was measured at different pH values; 2, 4, 6, 8, and 10; to better understand the stability of nanoparticles in the adsorption process. The zeta potential of nanocomposite showed a typical dependence on pH value, as shown in Figure 3A–C and Table S1. The nanocomposite displayed negative zeta potentials in the pH range from 2 to 10. This is related to the presence of different redox states of nanocomposite at different pH values. Protonation increases at low pH due to the production of radical cations and the binding of protons to the amine groups, decreasing negative zeta potential. The lowering of the negative zeta potential magnitude above pH 8 due to deprotonation of the amine groups of PPy resulted in less oxidation and decreased negative sites [41]. As predicted, the integration of PPy with silver nanoparticles results in a negative charge. As a result, the sample had a large particle size distribution (959 nm), as calculated by DLS measurements (Figure 3). This value is larger than that reported using SEM or TEM images, as illustrated in Figure 2. This is because the DLS technique permitted ZnO NPs to aggregate in an aqueous solution, while the SEM or XRD techniques do not allow for aggregation because the samples are measured dry.

#### 3.1.3. Chemical Functional Groups

**Figure 4:**
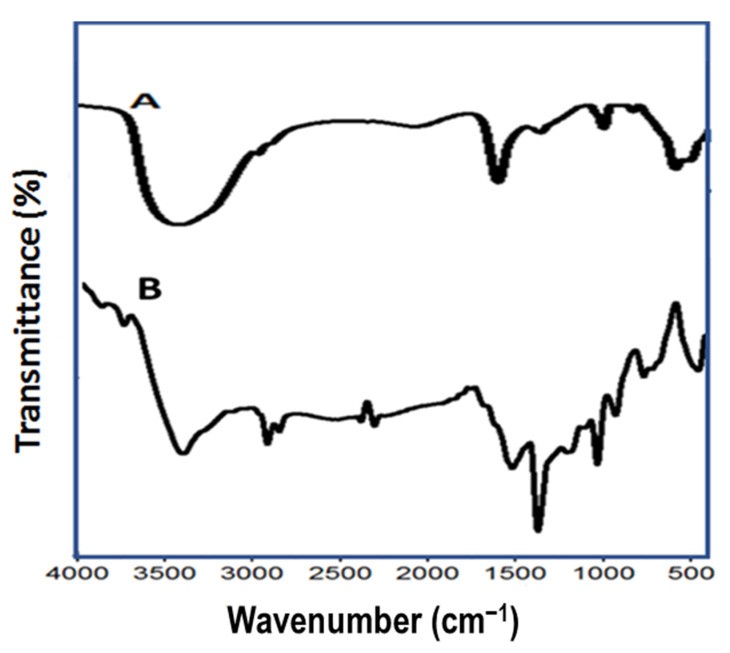
FTIR spectra of (**A**) ZnO and (**B**) ZnO–Ag/PPy nanocomposite.

The surface functional groups of ZnO and ZnO–Ag/PPy nanocomposite was examined by FTIR, as shown in Figure 4A. The FTIR spectrum of ZnO NPs showed peaks located at 620 and 540 cm^−1^, attributed to the Zn–O stretching modes [41]. The band at 3490 cm^−1^ is attributed to the stretching mode of O–H due to the water adsorption on the surface of ZnO NPs. The two bands at 1378 and 1633 cm^−1^ are attributed to the symmetric and asymmetric C=O bending vibrations, respectively.

ZnO–Ag/PPy nanocomposite, Figure 4B, displayed a band at 3490 cm^−1,^ which could be ascribed to the N–H stretching vibration of PPy. The modes associated with the symmetric and asymmetric stretching of -CH_2_ groups at 2992 and 2850 cm^–1^, respectively. The two peaks located at 1290 and 1478 cm^−1^ are related to C–N stretching vibrations of PPy. Modes at 1520 and 1164 cm^−1^ correspond to the stretching vibrations of the C–C and C=C backbone in the pyrrole ring [42]. The band at 1060 cm^−1^ is due to N–H wagging. The band in the region of 1000–850 cm^−1^ is attributed to the C–H wagging vibration of PPy [43]. The peak centered at 496 cm^−1^ is assigned to the metallic stretch of the silver [44,45]. These results indicate that the ZnO–Ag/PPy nanocomposites were successfully synthesized.

#### 3.1.4. Electrical Properties and Dielectric Constant

**Figure 5:**
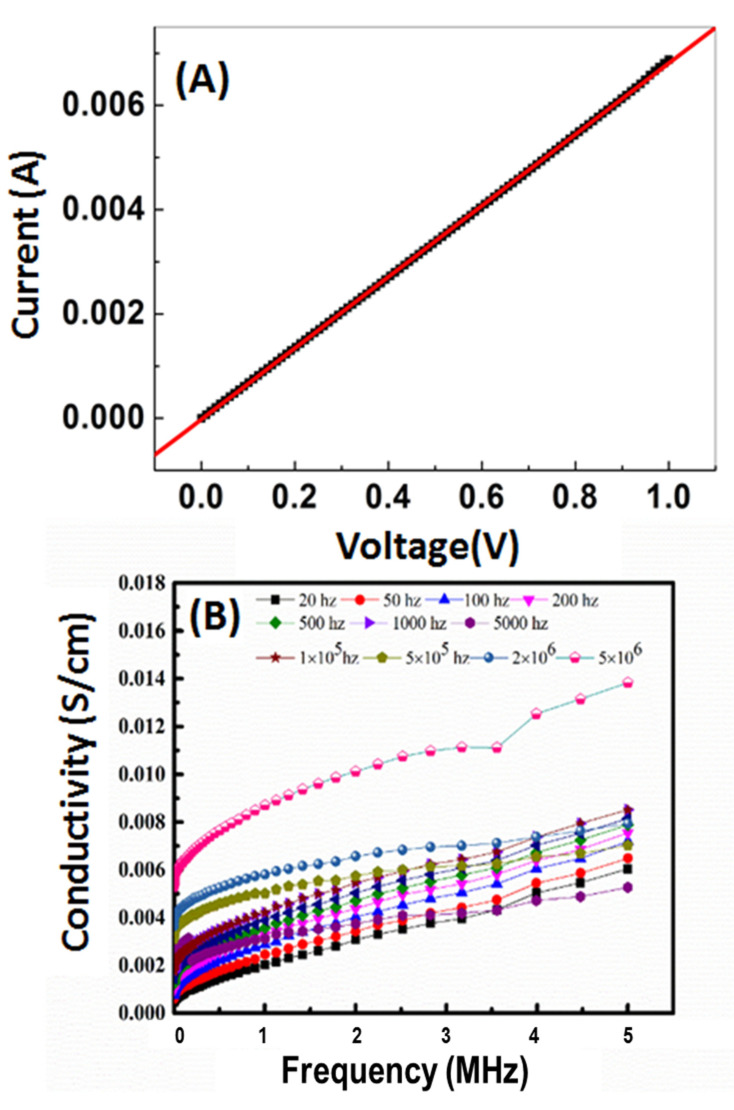
Electrical properties of nanocomposite; (**A**) direct current versus voltage and (**B**) frequency dependence of AC conductivity, σ_ac_(ω), at various temperatures.

The direct current (DC) versus voltage characteristics for nanocomposite was measured at room temperature using a Keithley measurement source unit and shown in Figure 5A. It is showing a good Ohmic behavior with average DC resistance of 1.415 ± 0.013 kΩ and DC conductance 700.280 ± 6.434 μS. The linear behavior may be due to two reasons; the first is the mobility of electrons during the chain of the nanocomposite, and the second one is attributed to the semiconducting nature of ZnO in the nanocomposite [46].

### 3.2. Frequency and Temperature Dependence of AC Conductivity

Figure 5B shows the dependence of AC conductivity (σ_ac_) on the frequency within a range from 20 to 5 × 10^6^ Hz (with 0.8 mm thickness) at different temperatures ranging from 283 to 298 K. The obtained results were represented by Equation (3):

σ_ac_(w) = Aw^S^ (3)

where A is constant, w is the angular frequency, w = 2πf, and S value is the frequency exponent.

**Figure 6:**
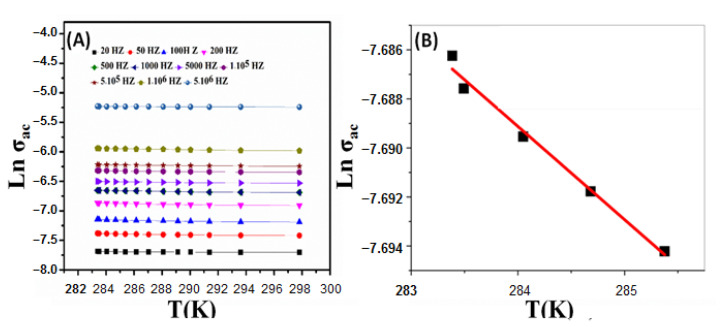
(**A**) variation of AC conductivity, σ_ac_(ω), with the temperature at different frequencies for nanocomposite and (**B**) variation of AC conductivity, σ_ac_(ω), with the temperature at a certain frequency.

**Figure 7:**
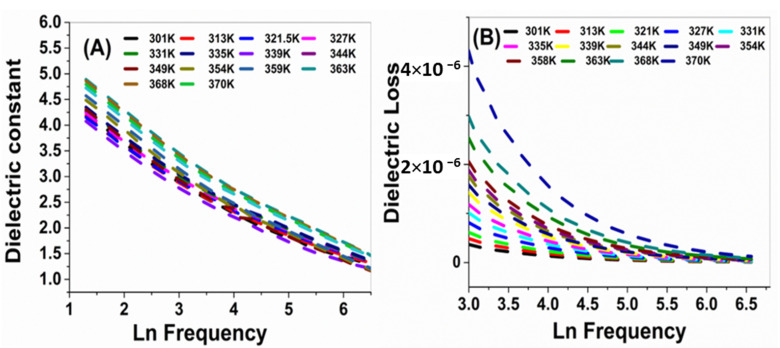
Dielectric analysis of nanocomposite; (**A**) dielectric constant and (**B**) dielectric loss as a function of frequency.

It was found that σ_ac_ was positively correlated with frequency at all studied temperatures, which may be due to the increase of the movement of the dipole molecular chains within the nanocomposite at high temperatures. In addition, the components of nanocomposite act as captures of the charge carriers, which are transported by the hopping process. Figure 6A shows the variation of ln(σ_ac_) as a function of T in Kelvin. The σ_ac_ at higher frequencies showed weak temperature dependence. The activation energy (ΔE_a_) is calculated at different frequencies from the slope of the straight lines in Figure 6B according to Equation (4);

σ_ac_(w) = σ_0_ exp(−ΔE_a_(w)/k_B_T) (4)

where σ_0_ is a constant, ΔE_a_ is the activation energy, k_B_ is the Boltzmann constant, and T is the absolute temperature. These results confirmed the electronic interaction between the electroactive metal centers (ZnO and Ag) and the polymer backbone. This can increase its electrocatalytic activity by improving electron transport in the polymer and lead to novel electronic and electrochemical properties [47]. The dielectric analysis measures the ac electric properties of a material as a function of frequency and temperature. Through this analysis, the dielectric constant (ε*′*) and dielectric loss (ε″) of a material can be determined. Figure 7A,B shows the variation of ε*′* and ε″ with the frequency at different temperatures. Figure 7A shows that ε*′* negatively correlated with frequency. The decline of ε*′* was observed due to the contribution of multicomponent of polarizability, deformational (electronic and ionic), and relaxation (orientational and interfacial) polarization at low-frequency. Similarly, ε″ dependence on the frequency at different temperatures is shown in Figure 7B [48]. This decrease may be ascribed to the migration of ions at low frequencies. Conversely, at low or moderate frequencies, the high values were due to the contribution of jumping ions to conduction loss during ions transfer, which is considered the main source of ε″ at low frequencies. Accordingly, the high values of ε″ at low and moderate frequencies are due to the contribution of ion jumping and conduction loss of ion migration.

### 3.3. Adsorption Properties

#### 3.3.1. Effect of Initial pH on Adsorption of Inorganic Ions (Cd^2+^, PO_4_^3−^) in Single and Binary Solutions

**Figure 8:**
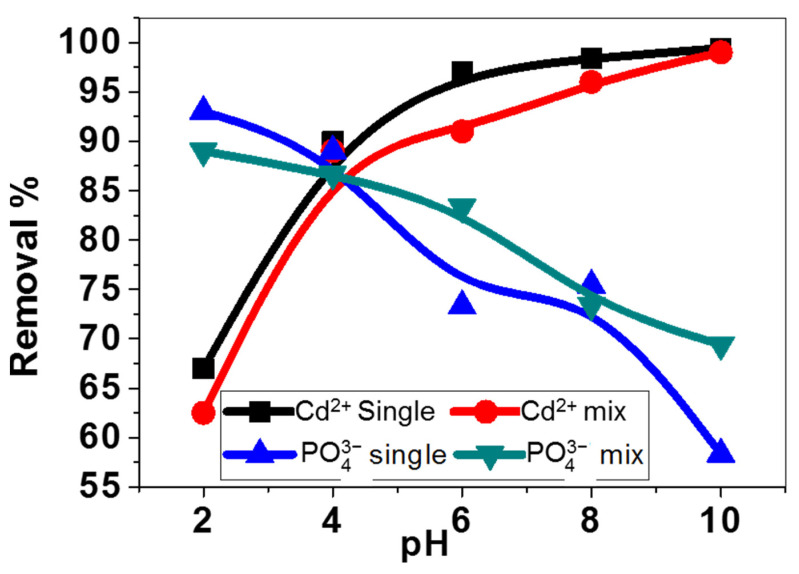
Effect of solution pH on the removal% in single and binary solutions.

pH is a vital parameter controlling the adsorptive removal of the metal ions in single or binary contaminates solutions (Cd^2+^ and PO_4_^3−^) due to the degree of dissociation among the functional groups in the adsorbent and variation of metal ions. Figure 8 shows the influence of pH on the adsorption capacity of the ZnO–Ag/PPy nanocomposite. It is evident that the adsorptivity of Cd^2+^ increases with increasing the pH value in the single system. This is related to the protonation of amine sites in PPy, which is involved in the adsorbent composite, which cannot assist the chelation process in an alkaline medium. However, with increasing pH value, the protons are discharged from the amine function groups of PPy adsorbent, which causes more accessible, dynamic amine destinations for the adsorption of Cd^2+^ ions from the contaminated solution. In addition, the adsorptivity of Cd^2+^ in binary contaminants system solution is increased with increasing pH value due to the selective adsorption of binary contaminants system solutions. The removal of cadmium ions can be accomplished at pH ≥6, which may be identified with the precipitation of Cd(II) ions as CdHPO_4_ at pH 6 and as Cd_10_(PO_4_)_6_(OH)_2_ at pH 7.5–8.0 [48,49].

The adsorptivity of our composite is increased from 62.5% to 99.2% as the pH increased from 2 to 10. This is ascribed to the negative value of the zeta potential of the composite, which changed from −5.2 mV to −28.2 mV as the pH increased. This generates an electrostatic attraction between the adsorbate and adsorbent, which increased with increasing pH value.

On the contrary, in a single system, the adsorptivity of PO_4_^3−^ diminishes with increasing pH value. This is attributed to the repulsion force between the surface of the composite and the contrarily charged species in the solution. While in a competitive environment (multiple contaminants system solution), the composite illustrated a higher PO_4_^3−^ absorptivity at pH value < 6 than at pH value ≥ 6, where the predominant species is H_2_PO_4_^−1^. Subsequently, the adsorption of phosphate and Cd^2+^ in the multi-contaminant framework may be improved by synergistic adsorptions among phosphate and Cd^2+^. However, at pH ≥ 6, the complex of phosphate and cadmium ions may be formed; thus, the adsorptivity of PO_4_^3−^ decreases.

#### 3.3.2. Kinetic Studies

**Figure 9:**
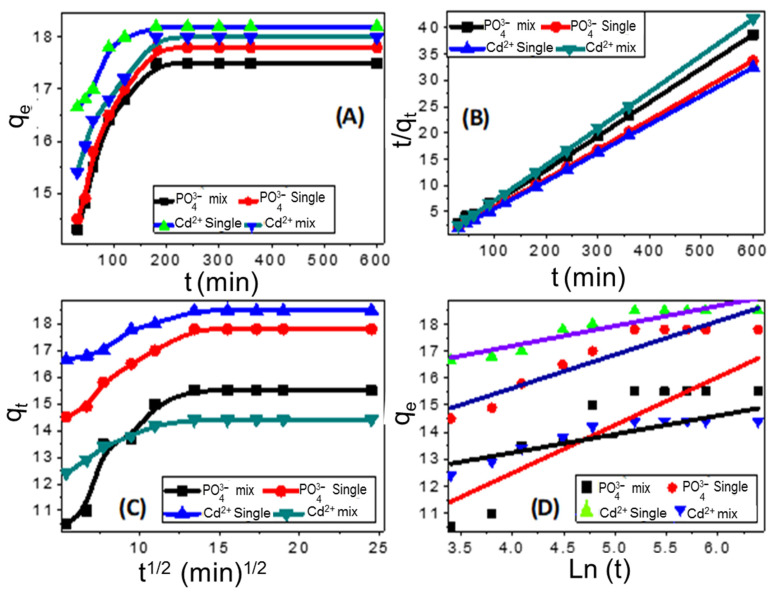
Kinetic studies of adsorption of Cd^2+^ and PO_4_^3−^ by nanocomposite; the relation between the contact time and uptake capacity (**A**), plot for the second-order model (**B**), plot for intra-particle diffusion model (**C**), and plot for Elovich kinetic model (**D**).

Figure 9A shows the adsorption behavior of the ZnO–Ag/PPy nanocomposite for cadmium and phosphate in single and binary contaminate solutions as a function of contact time. Clearly, the two adsorption stages in Figure 9A confirmed the noticeable increase in the uptake capacity with increasing the contact time in the first stage. While the equilibrium state for the adsorption systems represents the second stage. At the first-time equilibrium, all active sites that were vacancies become available for adsorption [37].

#### 3.3.3. Kinetic Models

Adsorption kinetics gives clear information about the adsorption mechanism. To study the kinetic process of the adsorption between the ZnO–Ag/PPy nanocomposite and cadmium and phosphate in single and binary contaminate solutions, pseudo-second-order, intra-particle diffusion, and Elovich kinetic models were employed. Based on the pseudo-second kinetic model, the chemisorption process between nanocomposite and ions (Cd^2+^ and PO_4_^3−^) was represented in the linear form in Figure 9B and Equation (5) as:

(5) $$\frac{t}{{\;q}_{t}}=\frac{1}{K_{2}q_{e}^{2}}+\frac{t}{q_{e}}$$

where *K*_2_ (g·mg^−1^·min^−1^) is the second-order rate constant, and the initial adsorption rate h (mg/g min) can be determined from h = *K*_2_q_e_^2^. The values of qe and *K*_2_ can be estimated from the slope and intercept of the linear fitting of Figure 9B.

To evaluate the function of the diffusion-controlled processes, the adsorption kinetics data were further analyzed using a linear form of the intra-particle diffusion model, which can be expressed as in Equation (6):

q_t_ = K_p_t^1/2^ + C (6)

Here, K_p_ is the slope of the linear portion, representing the intraparticle diffusion rate constant (mg g^−1^ min^−1/2^), and C is the intercept, which can be based on the thickness of the boundary layer. Two different linear regions were observed throughout the plot, which meant that the adsorption of Cd^2+^ and PO_4_^3−^ using nanocomposite involved more than one kinetic mechanism, as shown in Figure 9C and Table S2. The initial sharp gradient linear region (up to 5 min) could be attributed to the rapid surface adsorption of Cd^2+^ and PO_4_^3−^ ions controlled by boundary layer diffusion. The second linear portion of the plot (5–10 min) was attributed to intraparticle diffusion as the rate-limiting mechanism during this stage. Subsequently, the decrease in the concentration of solution slowed down the diffusion rate of the particles. Thus, reaching the final equilibrium stage (plateau stage from 14 to 25) represents the gradual saturation adsorption process [50]. Figure 9D and Equation (7) represent the linear plot of the Elovich model, which describes the chemical adsorption systems [37]:

(7) $$q_{t}=\frac{1}{\beta }\ln\left(\alpha \beta \right)+\frac{1}{\beta }\;\mathrm{In}\;\left(t\right)$$

where α parameter is the initial adsorption rate (mg/min) at reaction time t = 0 min, and the β parameter (1/slope) is the degree of surface coverage (g/mg). Figure 9D and Table S1 displayed the estimated parameters of the model from the linear plot curve between q_t_ and ln (t).

#### 3.3.4. Adsorption Isotherms in Single and Comparative Systems

##### Equilibrium Studies

**Figure 10:**
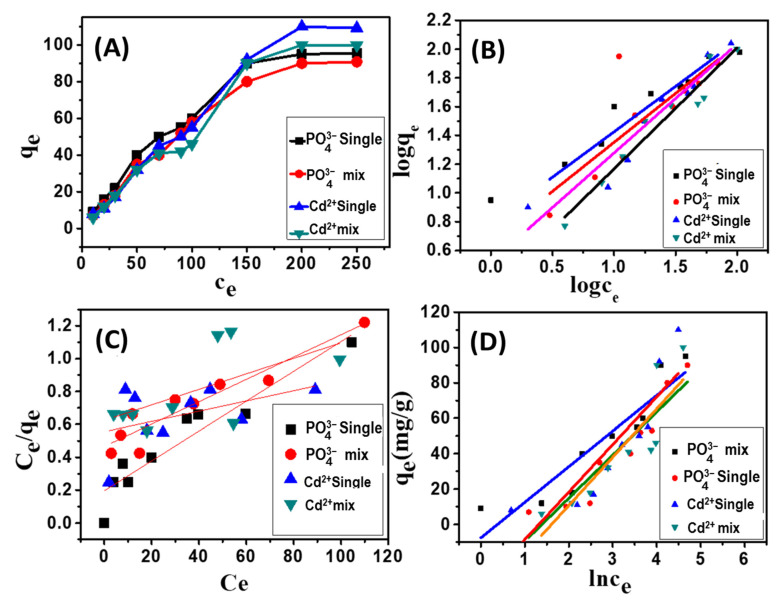
Equilibrium studies of Cd^2+^ and PO_4_^3−^ adsorption using the synthetic composite; effect of the initial concentration on the adsorption capacity (**A**), Freundlich isotherm model (**B**), Langmuir isotherm model (**C**), and Temkin isotherm model (**D**).

The isotherm evaluated the relation between the amount of adsorbed substance by an adsorbent (nanocomposite) and the equilibrium concentration of the ions at a constant temperature [51]. Regarding the effect of the different initial concentrations of pollutants on the adsorption capacity of the nanocomposite, Figure 10A, there is an increased adsorption capacity of all the studied pollutants with increasing the concentration of catalyst to reaching the equilibrium. The adsorption of Cd^2+^ in the single system increased from about 9 mg/g to about 90.7 mg/g, raising the metal concentration from 10 mg/L to 250 mg/L. While the adsorption of Cd^2+^ ions in binary systems shows a slight increase in the adsorption capacity from 40 to 85 mg/g, increasing the Cd^2+^ concentration from 75 mg/L to 150 mg/L. Similar adsorption results were detected for PO_4_^3−^ ions. In addition, for the effect of the different initial concentrations of pollutants on the adsorption capacity of the nanocomposite, there is increased adsorption capacity of all the studied pollutants by increasing the concentration of catalyst to reaching the equilibrium. The adsorption of Cd^2+^ in the single system increased from about 9 mg/g to about 90.7 mg/g, raising the metal concentration from 10 mg/L to 250 mg/L. While the adsorption of Cd^2+^ ions in binary systems shows a slight increase in the adsorption capacity from 40 to 85 mg/g, increasing the Cd^2+^ concentration from 75 mg/L to 150 mg/L. Similar adsorption results were detected for PO_4_^3−^ ions. In addition, the adsorption capacity of PO_4_^3−^ ions in the binary system increased from 10.2 mg/g to ~80 mg/g with rising the used concentration from 10 mg/L to 150 mg/L.

##### Common Isotherm Models

Different adsorption isotherms were employed in the adsorption of Cd^2+^ and PO_4_^3−^ on the nanocomposite surface, including Langmuir, Temkin, and Freundlich. The linearity and R^2^ values are the principal factors evaluating the most fitted model for the adsorption process. Langmuir model indicates the monolayer adsorbate on the homogenous surface adsorbent [51]. Its linear equation was expressed by Equation (8), as shown in Figure 10B:

(8) $$\frac{C_{e}}{q_{e}}=\frac{1}{bq_{max}}+\frac{C_{e}}{q_{max}}$$

where *C_e_* is the equilibrium concentration (mg/L), *q_e_* is adsorbed pollutant at equilibrium (mg/g), *q_max_* is the maximum removal capacity (mg/g), and *b* is the model isotherm constant (L/mg).

While Freundlich model hypothesizes that the adsorption process depends on the heterogeneous reaction, this model was expressed by Equation (9):

Log q_e_ = (1/n) log C_e_ + log K_F_ (9)

where K_F_ and n are the constants related to the ion adsorption efficiency and intensity, respectively.

The adsorption data of cadmium and phosphate ions in a single and binary system in Figure 10C displayed good agreement with the Freundlich model, where the determined coefficient value (R^2^) is more than 0.95. Table S2 showed the estimated Freundlich factor (1/n) is less than unity, which indicated the chemisorption of ions by the composite [47].

The third used model in the equilibrium evaluation of the studied adsorption system is the Temkin equilibrium model. This model involved a continuous interaction between pollutants and adsorbents [52]. This was expressed by Equation (10):

(10) $$q_{e}=B_{T}{\mathrm{InK}}_{T}+B_{T}\mathrm{In}\;C_{e}$$

where B_T_ is Temkin constant and K_T_ is a constant related to the equilibrium binding in L/g. Figure 10D and Table S3. Comparing R^2^ of these models, it can be concluded that the equilibrium sorption data of Cd^2+^ and PO_4_^3−^ in single and binary systems using nanocomposite were best described using the Freundlich isotherm model. The order of fitness of the isotherm models are Freundlich > Langmuir > Temkin. In addition, to K_F_ values of the Freundlich (1.1240 and 2.2261 L mmol^−1^ for adsorption of Cd(II) and Pb(II) agreed well with the experimental results. 1/n form Freundlich isotherm was less than 1, suggesting the SR-PAA hydrogel was favorable to adsorb of metal Cd(II) and Pb(II). Therefore, the analysis results showed that the Freundlich isotherm model was fitted the equilibrium Cd(II) and Pb(II) adsorption better than the Langmuir model. This refers to the multilayer adsorption of nanocomposite toward Cd^2+^ and PO_4_^3−^ ions. Therefore, the Freundlich model can describe the adsorption of all pollutants (Cd^2+^ and PO_4_^3−^ in single and binary systems) using nanocomposite. Fitting of the adsorption results of Cd^2+^ and PO_4_^3−^ with the Freundlich isotherm model describes the heterogeneous adsorption and the raising of the adsorption capacity, which causes an exponential decrease in the binding surface energy. This refers to the multilayer adsorption of nanocomposite toward Cd^2+^ and PO_4_^3−^ ions [13].

### 3.4. Antimicrobial Activity of the Composite

**Figure 11:**
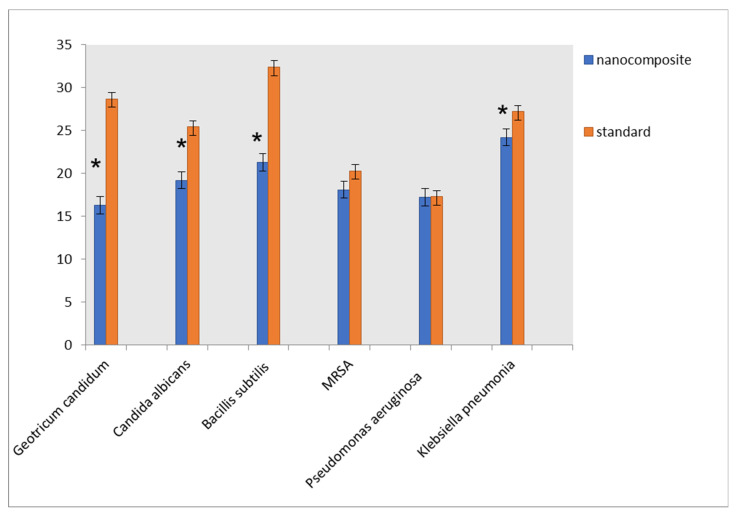
Antimicrobial activity and MIC of nanocomposite for different microorganisms. * studied composite is statistically significant from the standard at *p* values less than 0.05.

Hazardous microorganisms are considered one of the most severe problems of water pollution. Hence, dealing with this problem is an urgent issue. The nanocomposite could be evaluated for its antimicrobial activity against various Gram-positive bacteria, MRSA, Gram-negative bacteria, and fungi (Figure 11 and Table S4) using the agar disk diffusion method. Ampicillin, vancomycin, gentamycin, and amphotericin B were used as reference antibiotics. The nanocomposite showed high antimicrobial activity against all the tested microorganisms. This high activity may be ascribed to different factors, such as the release of Ag ions, interaction with the cell surface, and breakage of the cell membrane [53]. Additionally, the presence of ZnO nanoparticles in the nanocomposite matrix showed bactericidal effects on Gram-positive and Gram-negative bacteria as well as the spores [54]. ZnO reduces bacteria viability. In other words, the accumulation of the ZnO nanoparticles on the bacteria surface due to the electrostatic forces could be another mechanism of the antibacterial effect of ZnO nanoparticles [54]. Moreover, reactive oxygen species (ROS) are generated on the surface of the particles by nano photocatalytic processes. These species can penetrate the cell wall and hence damage the DNA and enzymes of bacteria and finally lead to the death of the microorganism [55,56].

Moreover, the interruption of electron transportation through transmembrane has been stated in the case of some metal nanoparticles, such as Ag and Zn [56,57]. The reaction of ZnO NPs with the cell of bacteria by a liaison of them to bacteria cell wall causing the killing of bacteria by rupture of their cell wall [58,59]. In addition, the release of Zn^+2^ with zeta potential = −15.7 mV can disturb all the metabolic processes of microorganisms through the electrostatic attraction with the negative charge of the polysaccharides of lipopolysaccharide, which predominate over the amide [59,60].

#### MIC Values of the Composite

Minimum inhibitory concentration (MIC) values of the nanocomposite against different species of microorganisms as bacteria and fungi are reported in Table S4. For gram-positive bacteria, the MIC values were 1.95 and 7.81 μg/mL for *Bacillus subtilis* and MRSA, respectively. The MICs values against *Pseudomonas aeruginosa* and *Klebsiella pneumoniae* were 15.63 and 0.49 μg/mL, but for fungi were 31.25 and 3.9 μg/mL for *Geotrichum candidum* and *Candida albicans*. These results refer to the higher activity of the nanocomposite toward bacteria than fungi. Although of their non-permeable cell walls, our nanocomposite showed the highest antibacterial performance against the gram-negative bacteria. This implies that the nanocomposite has a promising antibacterial effect. Compared with previous works, Table S5, our results achieved a dual effect for bioremediation of wastewater, adsorption of Cd^2+^ and PO_4_^3−^ in a single and competitive system. Our nanocomposite is used as a potent adsorbent for Cd^2+^ and PO_4_^3-^ions with high-efficiency > 95% for Cd^2+^ ions and >85% for PO_4_^3−^. Simultaneously, this nanocomposite exhibited strong antimicrobial activities against various types of bacteria and fungi. To the best of our knowledge, this is the first study that has developed a ZnO–Ag/polypyrrole composite with high electric properties utilizing microwave irradiation technique in the presence of the ZnO core side. Interestingly, the developed composite exhibited antimicrobial activity combined with the adsorption behavior of different pollutants. Simultaneously, the prepared ZnO–Ag/PPy displayed enhanced adsorption of Cd^2+^ and PO_4_^3−^ ions in single and mixed solutions. Table S5 illustrated the comparison between applying some previously reported PPy-based composites and our nanocomposite for adsorption of different pollutants and their antimicrobial activities. As shown in this table and until this time, there are no papers have been reported about using PPy-based nanocomposite with high dual ability in the bioremediation of wastewater and removal of Cd^2+^ and PO_4_^3−^.

## 4. Conclusions

This study demonstrated the preparation of multifunctional ZnO–Ag/PPy nanocomposite in the form of core/shell structure by using the microwave irradiation technique. The fabricated nanocomposite featured good electrical properties. Moreover, the synergetic effect between ZnO, Ag and PPy has been proved to contribute to the superior adsorption capacity of the ZnO–Ag/PPy core–shell hybrid toward toxic Cd^2+^ and PO_4_^3−^ in single and binary systems reaching >99% and 93%, respectively, at the optimized value of pH. The adsorption data of the ZnO–Ag/PPy adsorbent toward Cd^2+^ and PO_4_^3−^ ion fits the Freundlich isotherm and follows pseudo-second-order reaction kinetic model. Moreover, the prepared ZnO–Ag/PPy nanocomposite was proved to have a good antimicrobial performance for various hazardous microorganisms, such as fungi, Gram-positive and Gram-negative bacteria. It showed a high antimicrobial performance with MIC values ranged from 0.49 to 31.25 µg/mL. It is believed that the ZnO–Ag/PPy core–shell hybrids will find promising practical applications in terms of high adsorption capacity, high antimicrobial activity, easy availability, and low cost for water bioremediation.

## Data Availability

Not applicable.

## Supplementary Materials

The following are available online at https://www.mdpi.com/article/10.3390/nano11071688/s1, Scheme S1. Schematic representation for the method of formation of PPy–Ag nanocomposite; Figure S1. EDAX of ZnO–Ag-PolyPyrrole composite.; Figure S2. Particle size distribution from SEM images. Table S1. Zeta potential and size particle of the nanocomposite.; Table S2. Adsorption kinetic parameters of PO_4_^3−^ and Cd^2+^ onto ZnO-Ag /PPy composite.; Table S3. Adsorption isotherm parameters of PO_4_^3−^ and Cd^2+^ onto ZnO-Ag/PPy composite.; Table S4. Mean inhibition zone in mm ± Standard deviation beyond well diameter of 6 mm using 10 mg/mL concen-tration of the nanocomposite and minimum inhibitory concentration (MIC) values (µg/mL) of the nanocomposite against a range of environmental and clinically pathogenic microorganisms.; Table S5. The heavy metal adsorption and the antimicrobial response of previously reported PPy–based nanocomposite and ZnO/Ag nanocomposite compared to present ZnO-Ag/PPy.
