# Supplementary material for: Design and Characterization of a Novel ZnO–Ag/Polypyrrole Core–Shell Nanocomposite for Water Bioremediation
Source: Nanomaterials (Basel). 2021 Jun 28;11(7):1688. doi: 10.3390/nano11071688 (PMC8308129; doi:10.3390/nano11071688)
Supplement: Supplementary file 1 [file nanomaterials-11-01688-s001.zip › nanomaterials-1206628-supplementary.pdf]

Supplementary Material

Design and Characterization of a Novel ZnO–Ag/Polypyrrole Core–Shell Nanocomposite for Water Bioremediation

Fatma Mohamed [1,2], Abeer Enaiet Allah [2], Khulood A. Abu Al-Ola [3] and Mohamed Shaban [1,4,*]

[1] Nanophotonics and Applications (NPA) Lab, Physics Department, Faculty of Science, Beni-Suef University, Beni-Suef, 62514, Egypt; fatma.mohamed@science.bsu.edu.eg
[2] Chemistry Department, Faculty of Science, Beni-Suef University, Beni-Suef 62511, Egypt; abeer.abdelaal@science.bsu.edu.eg
[3] Department of Chemistry, College of Science, Taibah University, Al-Madinah Al-Munawarah 30002, Saudi Arabia; kabualola@taibahu.edu.sa
[4] Department of Physics, Faculty of Science, Islamic University in Madinah, Al-Madinah Al-Munawarah 42351, Saudi Arabia
* Correspondence: mssfadel@aucegypt.edu

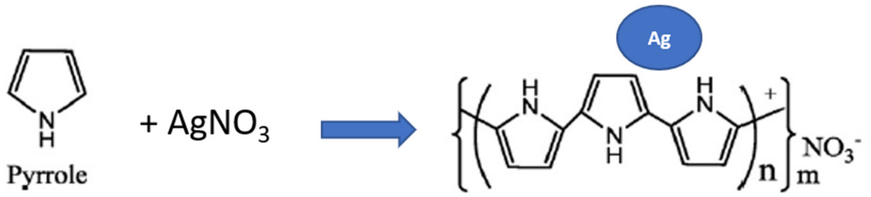

Scheme S1. Schematic representation for the method of formation of PPy–Ag nanocomposite.

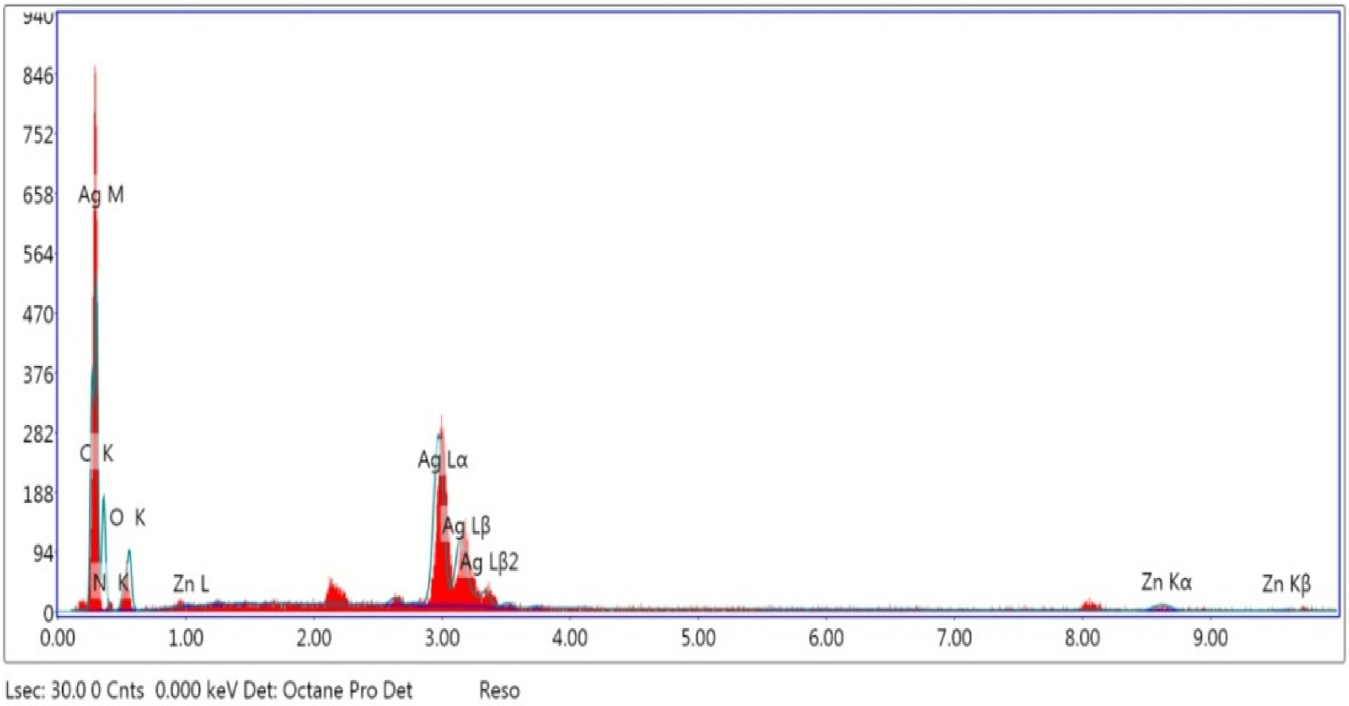

Figure S1. EDAX of ZnO–Ag-PolyPyrrole composite.

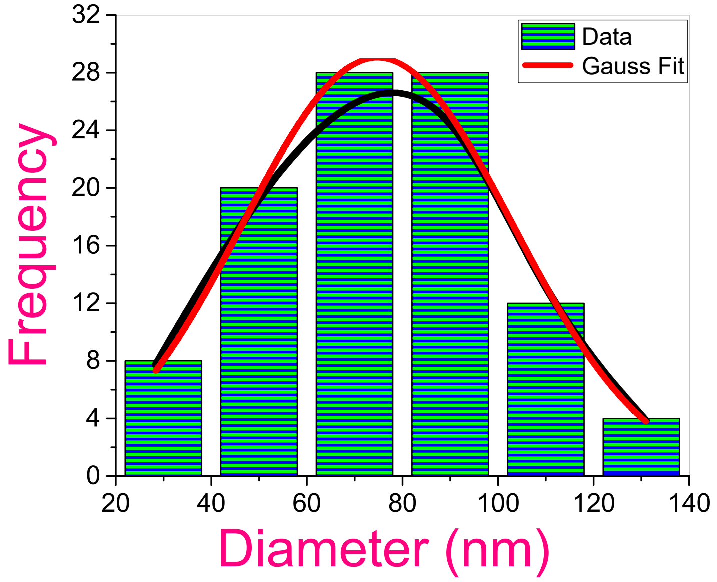

Figure S2. Particle size distribution from SEM images.

Table S1. Zeta potential and size particle of the nanocomposite.

Size with standard deviation; d ± SD (nm)	Zeta potential = −15.8 ± 5.98 mV
614 ± 196.4	Zeta deviation = −6.02 mV
4939 ± 651.1	Conductivity = 1.2 ms/cm

Table S2. Adsorption kinetic parameters of PO_4^{3-} and Cd^{2+} onto ZnO-Ag /PPy composite.

Model	Parameter	PO_4^{3-} binary	PO_4^{3-} single	Cd^{2+} single	Cd^{2+} binary
Pseudo second order	q_e	15.9	18.11	18.69	14.55
	K_2	0.00527	0.0077	0.0135	0.0168
	R^2	0.99913	0.99979	0.99997	0.99992
Elovich	β	0.563	0.806	1.3	1.4
	α	37.3	6097	$3.3x10^7$	$3.6x10^7$
	R^2	0.72	0.85	0.854	0.8022
Intraparticle diffusion	K_p	0.25	0.17	0.108	0.096
	C	10.8	14.4	16.4	12.6
	R^2	0.58	0.68	0.688	0.60

Table S3. Adsorption isotherm parameters of PO_4^{3-} and Cd^{2+} onto ZnO-Ag/PPy composite.

Model	Parameter	PO_4^{3-} single	PO_4^{3-} binary	Cd^{2+} single	Cd^{2+} binary
Freundlich	logKf	-0.18	0.88	0.09	0.15
	1/n	0.982	0.58	0.98	0.97
	R^2	0.982	0.976	0.966	0.96
langumir	Q	147.05	111.11	321.5	250
	b	0.014	0.045	0.0056	0.0064
	R^2	0.883	0.89	0.10	0.289
Temkin	K	0.76	0.277	0.268	0.2
	b	19.63	24.4	26.85	27.3
	R^2	0.866	0.87	0.726	0.74

Table S4. Mean inhibition zone in mm ± Standard deviation beyond well diameter of 6 mm using 10 mg/mL concentration of the nanocomposite and minimum inhibitory concentration (MIC) values (μg/mL) of the nanocomposite against a range of environmental and clinically pathogenic microorganisms.

Tested microorganisms	Inhibition zone (mm)	St.	MIC (μg/mL)	St.
FUNGI	Nanocomposite	Amphotericin B	Nanocomposite	Amphotericin B
Geotricum candidum (RCMB 05097)	16.3 ± 1.2*	28.7 ± 0.72	31.25	0.98
Candida albicans (RCMB 05036)	19.2 ± 0.63*	25.4 ± 0.63	3.9	0.98
Gram Positive Bacteria:	Nanocomposite	*Ampicillin*	Nanocomposite	*Ampicillin*
Bacillis Subtilis (RCMB 010067)	21.3 ± 1.2*	32.4 ± 1.2	1.95	0.49
		Vancomycin		*Vancomycin*
Methicillin-Resistant Staphylococcus aureus (MRSA 2658 RCMB)	18.1 ± 0.72*	20.3 ± 1.2	7.81	3.9
Gram negativeBacteria:	Nanocomposite	*Gentamicin*	Nanocomposite	*Gentamicin*
Pseudomonas aeruginosa (RCMB 010043)	17.2 ± 1.2	17.3 ± 0.63	15.63	15.63
Klebsiella pneumonia(RCMB01002 23-5)	24.2 ± 0.58*	27.2 ± 1.5	0.49	0.49

*The studied composite is statistically significant from the standard at *p* values less than 0.05.

Table S5. The heavy metal adsorption and the antimicrobial response of previously reported PPy–based nanocomposite and ZnO/Ag nanocomposite compared to present ZnO-Ag/PPy.

Type of polymer composite	Adsorption of different pollutants	Antimicrobial activity	Ref.
ZnO –Ag/PPy	>99% and 93% for Cd^{2+} and PO_4^{3-} in single and binary systems, respectively.	Antimicrobial activity against various types of gram-positive and gram-negative bacteria with ihibiations zones and MIC values<br>*Bacillis Subtilis:* 21.3 ± 1.2 mm ; 1.95 µg/mL<br>*Methicillin-Resistant S.Aureus:* 18.1 ± 0.72 mm ; 7.81 µg/mL<br>*Pseudomonas aeruginosa:* 17.2 ± 1.2 mm; 15.63 µg/mL<br>Klebsiella pneumonia: 24.2 ± 0.58 mm; 0.49 µg/mL	Present work
PPy	-	Higher antibacterial activity against S. pneumoniae and E. faecalis and S. aureus with inhibition zones of diameters 15.8 ± 0.58, 14.9 ± 0.63and15.0 ± 1.22 mm and MIC values of 62.5, 125, and 125 mg/mL, respectively	[1]
PPy/Ag nanocomposite	-	Antibacterial activity against E. coli, K. pneumoniae, and S. aureus with inhibition zones of 21.4, 23.8, 29.7 mm.	[2]
PPy/graphene oxide	The Langmuir capacity for mercury(II) adsorption = 400.0 mg/g at 300 K and pH 7	-	[3]
Polyaniline microstructures	The maximum uptake capacity of Cr(VI) ions (q_{max}) = 59 mg/g	-	[4]
PPy/Ag sheets	-	Antibacterial activity toward E. coli and S. aureus with inhibition zones of 7.20 ± 0.72 and 7.66 ± 0.58 mm after 24 h of incubation	[5]
PPy/calcium rectorite	The maximum adsorption capacity for Cr(VI) = 833.33 mg/g at 45 °C.	-	[6]
Ag-doped ZnO	-	Antibacterial activity against *Staphylococcus aureus* and *C. Albicans* with a maximum inhibition zone of 17 and 18 mm	[7]

References

1. Sayyah, S.M.; Mohamed, F.; Shaban, M. Antibacterial activity of nanofabricated polypyrrole by cyclic voltammetry. *IOSR J. Appl. Chem.* **2014**, *7*, 11–15. doi:10.9790/5736-07211115.
2. da Silva Jr., F.A.G.; Queiroz, J.C.; Macedo, E.R.; Fernandes, A.W.C.; Freire, N.B.; da Costa, M.M.; de Oliveira, H.P. Antibacterial behavior of polypyrrole: The influence of morphology and additives incorporation. *Mater. Sci. Eng. C* **2016**, *62*, 317–322. doi:10.1016/j.msec.2016.01.067.
3. Zhou, C.; Zhu, H.; Wang, Q.; Wang, J.; Cheng, J.; Guo, Y.; Zhou, X.; Bai, R. Adsorption of mercury(II) with an Fe_3O_4 magnetic polypyrrole–graphene oxide nanocomposite. *RSC Adv.* **2017**, *7*, 18466–18479. doi:10.1039/c7ra01147d.
4. Shaban, M.; Abukhadra, M.R.; Rabia, M.; Elkader, Y.A.; El-Halim, M.R.A. Investigation the adsorption properties of graphene oxide and polyaniline nano/micro structures for efficient removal of toxic Cr(VI) contaminants from aqueous solutions; kinetic and equilibrium studies. *Rend. Fis. Acc. Lincei* **2018**, *29*, 141–154. doi:10.1007/s12210-018-0673-z.
5. Chondath, S.K.; Poolakkandy, R.R.; Kottayintavida, R.; Thekkangil, A.; Gopalan, N.K.; Vasu, S.T.; Athiyanathil, S.; Menampa-rambath, M.M. Water–Chloroform Interface Assisted Microstructure Tuning of Polypyrrolee–Silver Sheets. *ACS Appl. Mater. Interfaces* **2019**, *11*, 1723–1731, doi:10.1021/acsami.8b18943.
6. Xu, Y.; Chen, J.; Chen, R.; Yu, P.; Guo, S.; Wang, X. Adsorption and reduction of chromium(VI) from aqueous solution using polypyrrole/calcium rectorite composite adsorbent. *Water Res.* **2019**, *160*, 148–157. doi:10.1016/j.watres.2019.05.055.

7. Swati; Verma, R.; Chauhan, A.; Shandilya, M.; Li, X.; Kumar, R.; Kulshrestha, S. Antimicrobial potential of ag-doped ZnO nanostructure synthesized by the green method using moringa oleifera extract. *J. Environ. Chem. Eng.* **2020**, *8*, 103730. doi:10.1016/j.jece.2020.103730.
